# Sources of Health and Disease in Communities

**Published:** 1836

**Authors:** 


### Art. V.—Sources of Health and Disease in Communities.

*The Sources of Health and Disease in Communities, or Elementary Views of Hygiene, illustrating its importance to Legislators, Heads of Families, &c.* By Henry Belinaye, Esq., &c. Boston, Allen and Ticknor, pp. 160.

A small work with the above title, fell into our hands a few days since, which contains many things worthy of notice, and hence we propose to lay a short review of its principal features before our readers. Our author informs us, in the commencement of his introduction, that he proposes, in his essay to investigate the influence of laws, institutions, habits, climate, etc., on the vigor and health of man; an enquiry both laudable and useful. This subject doubtless has not been pursued by medical writers with the earnestness which its importance demands. It is almost universally acknowledged that certain diseases are hereditary, yet there is but little said in relation to the best mode of preventing the formation of constitutional defects, or of preserving the offspring of diseased parents from the contaminating influences of their predecessors. Civilization and its attending consequences undoubtedly has a great influence over the health of the human family. As society becomes restrained and complicated; as the luxuries of life increase, and as indolence prevails, the constitution becomes enfeebled and the muscular development greatly retarded. Many, and indeed most of our diseases were unknown to our aboriginal inhabitants, whose appetites and habits were solely the offspring of simple nature. It would be wise in us, therefore, to enquire how far the luxuries and habits of civilized life tend to shorten it, and to apply if possible a corrective at as early a period as possible. Even where legislative enactments are necessary they should be resorted to fearlessly, for it is every where acknowledged that no state can be powerful whose inhabitants are either mentally, morally or physically enfeebled. A sound population constitutes the strength of any nation. In the United States the habits of the people have made but comparatively few inroads upon their bodily developments, still we have no evidence that this state of things will continue. As the population becomes more dense, as wealth is accumulated in the hands of the few, and the many are shut up during the entire day in crowded manufactories or mines, or worn to death by the toils necessary to procure a bare subsistence, the frame must gradually be still more enfeebled through various succeeding generations. It is a common observation in our country that men of talents and persevering useful industry, in the professions or among statesmen, spring from those accustomed to a country life, where the various luxuries and dissipations of cities are comparatively unknown.

The growers of stock are aware of the influence which the parent has over the constitution and form of the offspring, and hence they exclude defective animals from their herds, and in this way, with various crossings, procure a race much more valuable in every respect than the root from whence it sprang. It would be difficult to do this among men by legislation, still if public opinion was properly enlightened on the subject, intermarriages among scrophulous families would be less frequent, and hence life would be prolonged and society proportionably benefitted. In relation to the effects of longevity upon a nation our author makes the following just remarks:

"Since it is certain, that every stage of human existence has a peculiar office assigned to it, as well as every instant a duty, it is highly important man should attain a certain degree of senility. Should the human being die in infancy or childhood, the loss to the state would not be great; but later, it is far otherwise. The business of adolescence is to acquire knowledge by example, and by memory; of the adult to apply this information; and later, to invent. At fifty, men begin to perfect and classify knowledge; to instruct and guide their fellow-creatures. Now, it is clear, that a state cannot advance so rapidly in civilization, where the average duration of life is short; and will be great in proportion to the approach of the majority of its subjects to sixty-five—an age that allows of acquisition, application, invention, and arrangement of the stores of knowledge. We must venture to repeat, that a man dying at eighteen or twenty, has only lived to *consume* the resources of society, and can leave nothing behind him, but a legacy of poignant regret. If he live till he has applied and invented, it is of the highest importance he should be allowed time to mature and consolidate what, as his own acquisition, he best understands. One able man may, perhaps, do more from the age of thirty-five to sixty-five, than twenty more coming after him, and dying successively at thirty-five, thirty-six, and thirty-seven, and so on to sixty-five. We must not forget likewise, that there is a period anterior to death, which is incapable of useful effort of mind and body; and (however short the rate of existence in any nation) some years of useless dotage will generally precede death.

"We must set a still greater value upon longevity, if we add to what has been stated, the sagacious remark of an eminent writer—that the greater mortality in southern climates before the age of thirty, is the reason that northern nations have invariably conquered those of the south."

From the remarks of our author on the subserviency, of man to physical influences we extract the following—

"In all parts of the globe, men have thought they recognized lunar influences on the crisis and exacerbations of disease. The phenomena of the invasion and duration of fevers, appears to be controlled by that influence. This is seen more distinctly as we approach tropical climates, where generally the operations of nature partake of a development very favorable to scientific investigation. Dr. Jackson states that in Jamaica, the febrile intermittent and lunar periods correspond. Dr. Lind observed the same phenomenon, and adds, that deaths occur mostly during the ebb of the tide; and that eclipses produce dangerous relapses in those ill of fever. Dr. Balfour, in the Asiatic Researches, says, that when the sun, during the equinoxes, by approaching the equator, increases, by his power, the attraction of the moon, with the increase of the tides occur also the increase of violent fevers, and mortality.

"By the variation of the relative position of the sun in the ecliptic, are produced the seasons; and by the seasons is the human frame greatly influenced. Statistical reports prove that the incipient life of the human being, as well as its death, are subject to their power.

"Conception, in woman, does not take place with equal facility at all periods of the year; the difference is marked, although it may be modified by climate. The same laws are only more inflexible in animals, because the circumstances of passion, and artificial temperature, are not, as with men, at their own command. However, the lower classes of society, whose propensities and whose circumstances bring them nearer to a state of nature, are more decidedly governed by the law of preference as to seasons. The end of the spring, and the beginning of summer, are the periods that in our climates tend, in a remarkable degree, to the increase of population. Taking for our guide the more correct reports of France, the most prolific months, drawn from accurate registers, may be arranged, according to the order of the relative number of births in each, thus:—March, January, February, May, August, October, September, July, November, June, and December. Since, then, the greatest number of births occur in March, January, and February, it must be obvious that the months most favorable to conception are, June, April, May."—

From these remarks it would seem that our author was a firm believer in the influence of the moon, and planets over health and disease. Some of his remarks strongly remind us of a portion of a lecture delivered by a professor in one of our Western Medical Colleges, in which it was stated that the greatest number of deaths always occured either at the new or full moon, or at the time of quartering, *or within five or six days of one of these periods.*

Under the head of laws of propagation we find the following, which though perhaps not entirely correct is nevertheless worthy of notice.

"The physical improvement or degradation of the race of man, like that of animals, appears to work, or revolve, chiefly on this one great principle—inheritance. Let philosophers revolt at the doctrine of the inheritance of evil, of which religion establishes the belief—or at that of property, which legislation has consecrated—it is certain, that inheritance, whether of good or of evil, is the universal law of nature. If we look at a succession of men, nearly related—like the first series of the Roman emperors, so often cited—we find, if we may venture on the comparison, the same resemblance of feature (in their statutes,) and of disposition (recorded in history,) that an Arab will show in the pedigree of a favorite horse. The noble features of better princes—those, for example, of the house of Lorraine, now reigning—no less resemble the portraits of their ancestors. Singular tribes of men, like the gypsies, have roamed throughout the world for centuries, unchanged in their lineaments, in spite of their intercourse with so many different nations—because habits, or laws, kept their marriages exclusively within their own caste.

"Union of individuals of different races, improves the breed, and, as we shall presently repeat, increases the number of male offspring—a circumstance which is always a proof of vigor, and a source of superiority. An abhorrence, we might call instinctive, pervades mankind with regard to marriages betwixt near relatives. Although at first prevalent, from necessity, for the increase of the species, it has justly fallen under the anathema of the canon law, for it tends to the degeneracy, physical as well as moral, of mankind. Some tribes still inhabit the earth, among whom marriages betwixt near relatives take place; their women are comparatively barren, and their progeny dwarfish. If we select similar examples in modern times, either in this, or in Catholic countries, where dispensations are granted for marriages betwixt persons nearly related; or if we refer to passages in ancient history—to the age of the Ptolemies, for instance—we find they no less generally afford grounds for animadversion.

"But in extending the same principle to any comparatively numerous, though still exclusive, class of society, the same remark holds good. We may perhaps explain from the sterility thus produced, combined with another cause we shall presently come to, the small proportion of families of elevated rank, that remain in Europe from feudal times, though their alliances and pedigrees were so honorable, and so carefully recorded. Towards the latter ages of feudalism, in the height of their aristocratic grandeur, a class of barons would marry only among their own kindred, and sterility struck them—whilst, in the earlier periods of nobility, accessions were constantly being made to that rank, by intermarriages with bold soldiers, who had earned their spurs in the field. Were it not illiberal to attack whole classes of men, among whom are found some noble exceptions to the rule, we might specify the aristocrary of a now isolated country, among whom this exclusive pride, and constant marriage of caste, have degenerated their race, from one of the finest in Europe, to one of the most puny, in size and in intellect. We may perhaps fairly ascribe to the same cause, the imbecility which has been so fatal to some former royal dynasties; certainty, if the laws of nations, in consecrating the great and useful principle of legitimacy, had prohibited the alliance of marriage of princes in any near degrees of relationship to each other, they would have infused into it a principle of self-preservation. Now, differences of religion, and political tenets, often confine royal marriages within a destructive sphere."

On page 37 we have the following just remarks:

"The Christian religion, by consecrating monogamy as the law of the faithful, not only promoted the surest means of civilization, but also of the increase and physical improvement of the human race. Marriage, thus hallowed by the most enlightened of systems, produces, in its turn, all the elements necessary to the civil governments of states, and to their physical strength—an effect which no other union of the sexes can achieve. But to obtain these results, there must be a freedom of choice, and certain physical conditions, observed in the conjugal compact, most of which fall within the scope of our particular views.

"Recent inquiries appear to lead to the conclusion, that, according as the strength predominates in the father, or mother, the child will be male or female, and that infirm fathers generally give life to female offspring. It would appear also, that in the great number of those hot countries where polygamy obtains, the number of female children greatly predominates, the fecundating power of the man is too much divided—his wives are sterile, or have a larger proportion of female issues. The proportion so general and useful, in Christian countries, of twenty-two men to twenty-one women, does not exist there. While all efforts towards civilization are abortive, and the influence of woman on society is lost, it is not strange to see such communities remaining, as they do, for ever barbarous and stationary.

"Should our remarks on the birth of females from debilitated fathers, appear to want confirmation, we may appeal to the analogy of comparative physiology. In the animal kingdom, do we not see more cows, ewes, hens, etc., than corresponding males of the respective species? and the reason is to be found in those habits that correspond to polygamy among mankind.

"A reason has been before stated for the extinction of great families—the facts first mentioned may explain, perhaps, why they so often terminate in one female scion.

"As it is often an object of importance, even in a political point of view, to perpetuate a race threatened with extinction, there are provisions made by the immutable laws of nature, to which resort may be had, whereby the probability of male offspring may be restored, and which should be allowed to supersede the objections raised by prejudice and ignorance: they have been sufficiently pointed out in this chapter.

"The decline of states, as well as of families, may be generally traced to the same source; after the exclusive pride which deteriorates, supervene the luxury and licentiousness which extinguish, a race of men—reducing them gradually from a noble and warlike community, to a diminutive and effeminate set of beings, obliged to trust for their defence to hirelings, until they become eventually the vassals of their own former slaves, or of stronger invaders."

It is more than probable that very early marriages are productive of much evil to the offspring of those who indulge in them. A whole race might be much enfeebled both in body and mind, if not entirely obliterated by a chain of premature connections. Many of the French writers have recorded facts which prove that such is the case. On this subject the little work before us holds the following language.

"There can be no question of the injurious effect of very early marriage, both upon the married couple and their offspring. We have the more palpable and best known examples of such results, in the short-lived and imperfectly civilized nations inhabiting warm climates, and in the marriages of princes in Europe. Having obtained a dispensation from the Bishop of Tours for that purpose, Lewis the Eleventh, not fourteen, cohabited with his queen, twelve years of age. A writer has inquired, whether this early initiation in the rites of marriage was not the cause of this sovereign's well-known defects in after time. We might also ask, whether it was not also the cause of the debility and early death of his son, Charles VIII.

"As the stage of complete physical development of each individual differs according to so many circumstances, the case of each individual will require separate consideration. The question must remain more for parental authority than for the law to decide, and the former should be enlightened by the advice of competent science. Certain conditions premised, to prevent an imprudent increase of population, the evil most pressing is, perhaps, on the contrary, that of late marriages. Every man owes it to society, not to contract the responsible task of paternity, first before his physical powers and his moral experience are sufficiently developed; and next, before he has acquired a sufficiency of fortune; but procrastination beyond that period is productive of serious evils. The early life of man is scarcely ever free from that promiscuous intercourse that injures his frame, and society is burthened by the infirm offspring of too tardy a moral reform, and of premature or positive old age.

The frames of the female part of the community begin at a certain period to assume more and more of rigidity unfavorable to the office and function of maternity; they should, therefore, be married before they have lost their natural plasticity. We might adduce facts from comparative physiology, tending to prove, on the contrary, that the circumstance of having previously born well-formed children, maintains in the organs of the female that mould of form that will enable them to have offspring of the same character on any new union with a different person, and one not favored by nature."

As medical men are bound by their various duties both to themselves and society to preserve the health of their fellows, as well as to relieve them if possible when in distress, they should exert all their powers against premature marriages.

They certainly are the means of preventing the perfect development of both body and mind, as well as the cause of the debility and early death of an imperfect offspring.

The next chapter we shall notice treats of emanations. These are sometimes pleasant and heathful, and at others disagreeable and pernicious. The odor of hyocyamns, produces a disposition to quarreling, while that of many of our garden flowers has the opposite effect. Our author mentions the poisonous effluvia which is said to arise from the Indian Upas as a proof of the deleterious effects of certain emanations, but the touching tales so ofter told of this tree are undoubtedly fabulous. Still we are aware of the injurious results of many emanations on individuals and communities. It is more than probable that most of our epidemics depend upon this cause. We quote the following from the remarks of our author on this subject.

"The curious and powerful effect of gases on the human economy, is well worthy of study. Their palpable influence is easily felt, since at one time we see the most robust persons, when exposed to a particular emanation, thrown into a fatal lethargy—others, of the most serious character, when they have breathed another gas, thrown into convulsions of laughter, and performing the most grotesque antics. B ıt emanations are often acting as invisible enemies upon our health and spirits, when we are least aware of them or their influence ; and if we have not discrimination to discover, and knowledge to enable us to remove the cause, vainly shall we resort to every other remedy for our distress.

"We shall not expatiate on several other emanations, comparatively unimportant, but no less curious, and in some instances of beneficial operation on the human frame—such as those arising from fresh meat, and other articles of food, to which our butchers and victuallers are supposed to be partly indebted for their portliness and good looks—singular instances, if well-founded, of the controul exercised on our bodies by surrounding media."

In the chapter on effluvia we find many assertions of a doubtful character. Indeed one of the principal defects in the book-makers of the present day is that they take too much on trust. They do not enter upon a special examination of all their doctrines by varied experiments. This should be done in every instance when practicable, but especially in such as are of a doubtful nature. In the chapter referred to, we find it stated that the gastric juice will digest the tissues of the stomach after death. Now although we are well aware that this statement has been made again and again by writers, still our own observations are opposed to the truth of the assertion. We have frequently examined the stomachs of animals after they had been mechanically deprived of life without detecting any lesion whatever, in their coats. We believe in the cases referred to by authors, that the lesions of the stomach existed before the death of the animal.

On the subject of animal effluvia we present our readers with the following remarks:

"Bad or little food, fatigue, want of air, light, or of any other element upon which vitality depends for its support, will, by diminishing its conservative power, abandon the body to the inroads of putrescency. Hence, from the moment it begins to droop, its effluvia become more and more acrimonious, until death deliver it up entirely to the ravages of putrescency. It is when vitality is affected, that the body throws out the most noxious effluvia, and it is particularly open to receive the germ of every foreign contagion. Thus, the virulence of one cause conspiring with that of another, it spreads through all classes of the community the disastrous and awful consequences of morbid influences. Such of the lower classes as are most subject to depression of vital power, afford a permanent hot-bed to the habitual contagions of each country, and an inviting resort to others, which either arise spontaneously, or are conveyed from distant lands by the intercourse of man, and add so awfully to the sum of disease and mortality.

"Could, then, the vigor of the lowest classes of society be maintained, not by luxury, but by an equable supply of the necessaries of life, earned by regular labor proportioned to their strength, epidemics and contagions would either sweep by innocuous, or prove comparatively harmless. This must be an additional incentive to the paternal care of governments, and to the charity of the affluent classes. Without this, legal enactments, whether for exterior or inferior protection, must prove comparatively nugatory, dealing, as they do, with a power that travels on the wind, or is generated with most irresistible virulence, in the obscurest corner of a dungeon, or in the lowest abode of human misery. Without this, in vain will the affluent classes fence themselves round, and lock themselves up from the ordinary intercourse of the community. They will but have increased the sum of misery, and at the same time the momentum by which the disease will be enabled to reach them. Health and disease are in immediate connection, as we have proved elsewhere, with good or bad government, general prosperity, or partial affluence. Nor should we forget, that selfishness affords no escape. Liberal ideas on these subjects are the result of civilization, and generally speaking, the civilization of the globe may be measured by its tables of mortality."

Although we do not concur with our author in all these remarks still many of them are worthy of notice. We are aware that any thing which depresses the powers of life favors the development] of disease, but we do not believe this to be the result of putrefaction. During our visitation by cholera, fear and grief, were great exciting causes, and it is to these depressing passions that we are to attribute the death of so many individuals in families of timid and affectionate individuals.

In relation to premature burials we extract the following paragraph.

"The record of all who have been buried alive, in all countries and ages, would form a fearful volume, and strongly guard us against a too hasty presumption of death. Even in the time of Pliny, alarm had begun to be felt on this subject; and he dedicates a whole chapter to it. Bodies have been found in burial-vaults, which had turned upon their faces or sides—which had bled—which had marks of self-inflicted violence upon them, etc. etc."

It is more than probable that cases occur, perhaps more frequently than we are aware, of burials before life has entirely ceased. Bruhier of the continent of Europe, observes that "out of one hundred and eighty examples of persons erroneously supposed to be dead, fifty-two had been buried alive, some had been opened after supposed death, fifty-two had spontaneously revived, after being put into their coffins. Seventy-two were discovered to be alive after having been deemed dead."

Our author very correctly condemns the practice of burying the dead within the precints of a city or within the walls of churches in country places. The effluvia arising from dead bodies is often destructive. We once lost a fellow student by a disease that resulted from the dissection of a dead body, which had remained for several months in a vault, and was brought to the dissecting room by mistake. The whole class amounting to five or six were sick, and one died after a confinement of several days. The odor was not strong, but it was so peculiar that we imagine that we can still *feel* it, even at this late period.

We fully concur in the remarks of our author on the importance of bathing, but we cannot give them to our readers for want of room. Ventillation is also a subject that requires strict attention, and one that should not fail to receive the notice of the profession. Much injury is frequently done to the public health by closed churches, school-houses, and other crowded places during both warm and cold weather.

We shall close our remarks on this little work by a quotation from the chapter on the influence of civilization on the health of man.

"As nations become less affluent, and recede from that state of society most favorable to the sciences and arts, morbid evils resume their sway; and it has been justly observed, that Egypt, which has already furnished us with another illustration, could not have had that flourishing and dense population, its unparalleled antiquities prove to have existed, if the plague formerly raged in that country, as it does at the present day. It has been clearly proved that the varied operations of a numerous population on the soil, etc., can alone banish disease from insalubrious countries.

"When we seek to add conviction to reasoning and probability, on the subject of the benefits of civilization, in our acceptation of the term, we find it proved, by the comparative duration of life in the present and in ancient times.

"The average duration of life amongst the respectable classes in cities, is in France, forty-two, in England, forty-five years. Among the patrician order in ancient Rome, the average was thirty years—giving to moderns the advantage of twelve years more of life in the one, and fifteen years in the other instance, over this great nation of antiquity. That no doubt may remain of this superiority being attributable to improved civilization, we have but to refer to the data of Dr. Vilerme, and compare the rates of mortality in France, at different periods of time: we shall ever find longevity increasing in proportion to civilization. The mortality in Paris, in the seventeenth century, was one in twenty-five or twenty-six, whilst in the fourteenth century, it was one in sixteen or seventeen. It is now one in thirty-two!"

